# Supplementary material for: Complexome profiling of the Chlamydomonas psb28 mutant reveals TEF5 as an early PSII assembly factor
Source: Plant Cell. 2025 Mar 17;37(6):koaf055. doi: 10.1093/plcell/koaf055 (PMC12164590; doi:10.1093/plcell/koaf055)
Supplement: koaf055_Supplementary_Data [file koaf055_supplementary_data.zip › psb28_interactive.zip!/plots/intensity/qualityPlots/peptides/ASA2_Cre09.g415550.t1.2.html]

### Cre09.g415550.t1.2

Trivial name: ASA2  
SeqCoverage [median]: 50.00  
Mass: 48.41 kDa  
  
 

| Sequence | MissedCleavage | Mass [Da] | wtIntensity | muIntensity | Score |
| --- | --- | --- | --- | --- | --- |
| AAAVLGAIK | 0 | 812.51 | 18924.55 | 26718.50 | 124.37 |
| AAAVLGAIKK | 1 | 940.61 | 48847.18 | 79789.90 | 151.29 |
| AASAGLVAK | 0 | 786.46 | 98225.95 | 132818.97 | 173.28 |
| AVPGLSAMADK | 0 | 1058.54 | 47184.85 | 58841.84 | 119.07 |
| AVPGLSAMADKLSR | 1 | 1414.76 | 24392.24 | 34011.17 | 100.11 |
| DGAAALLGAAAAAVQK | 0 | 1396.77 | 2328.83 | 0.00 | 129.89 |
| DVADAAVALAYLGTR | 0 | 1504.79 | 20922.54 | 15253.92 | 214.81 |
| EAAQALWGLAALR | 0 | 1368.75 | 1269.86 | 26119.13 | 163.90 |
| GFGLAVEHGR | 0 | 1041.54 | 31133.77 | 28416.80 | 190.73 |
| KAVPGLSAMADK | 1 | 1186.64 | 5271.66 | 9075.86 | 105.25 |
| KAVPGLSAMADKLSR | 2 | 1542.86 | 861.49 | 1304.06 | 55.01 |
| LLWGFGAAGVQDGK | 0 | 1417.74 | 25480.53 | 45226.06 | 206.97 |
| RVPDAALAGALTK | 1 | 1281.74 | 51944.57 | 122751.41 | 116.96 |
| SGAADVELFAAVTELAAAR | 0 | 1860.96 | 477.06 | 0.00 | 60.53 |
| SLSPAQVSFAVEAVGK | 0 | 1588.85 | 36379.16 | 64349.10 | 273.81 |
| SSAAAAFHAELAEAVAYASGAR | 0 | 2120.03 | 1536.84 | 0.00 | 52.54 |
| TADFKAADLAR | 1 | 1177.61 | 1672.57 | 9541.63 | 125.28 |
| TLAELAGPLAASLK | 0 | 1353.79 | 57704.42 | 172261.86 | 237.53 |
| VASFVSSGPDGSTLDVVVPADANTK | 0 | 2432.21 | 39093.85 | 80638.29 | 156.11 |
| VAVVPQTEFPTGAPLK | 0 | 1652.91 | 126500.58 | 219806.91 | 179.35 |
| VAVVPQTEFPTGAPLKQR | 1 | 1937.07 | 8787.44 | 23119.55 | 108.34 |
| VPDAALAGALTK | 0 | 1125.64 | 10132.67 | 37136.24 | 145.55 |
| VREAQGFK | 1 | 933.50 | 6246.63 | 10406.38 | 126.62 |
|  |  |  | 665319.2 | 1197587.6 |  |
